# Adaptation and implementation processes of a culture-centred community-based peer-education programme for older Māori

**DOI:** 10.1186/s43058-022-00374-3

**Published:** 2022-11-24

**Authors:** Mary Louisa Simpson, Stacey Ruru, John Oetzel, Pare Meha, Sophie Nock, Kathrine Holmes, Hariata Adams, Ngapera Akapita, Marama Clark, Kawarau Ngaia, Reuben Moses, Rangimahora Reddy, Brendan Hokowhitu

**Affiliations:** 1 Waikato Management School, University of Waikato, Hamilton, New Zealand; 2 University of Waikato, Hamilton, New Zealand; 3 Rauawaawa Kaumātua Charitable Trust, Hamilton, New Zealand; 4 Faculty of Māori and Indigenous Studies, University of Waikato, Hamilton, New Zealand; 5 Ranolf Medical Centre, Rotorua, New Zealand; 6 Te Korowai Hauora O Hauraki-Thames, Thames, New Zealand; 7 Ngāti Ruanui W’anau Ora, Hāwera, New Zealand; 8 Poutiri Charitable Trust, Te Puke, New Zealand; 9 Te Korowai o Ngāruahine Trust, Hāwera, New Zealand; 10 Te Roopu Tautoko Ki Te Tonga, Dunedin, New Zealand; 11 University of Queensland, Brisbane, QLD Australia

**Keywords:** Indigenous, Co-design, Culture centred, Community-engaged research, *Māori*, Health inequity, Implementation frameworks

## Abstract

**Background:**

Health inequities experienced by *kaumātua* (older Māori) in Aotearoa, New Zealand, are well documented. Examples of translating and adapting research into practice that identifies ways to help address such inequities are less evident. The study used the He Pikinga Waiora (HPW) implementation framework and the Consolidated Framework for Implementation Research (CFIR) to explore promising co-design and implementation practices in translating an evidence-based peer-education programme for older Māori to new communities.

**Methods:**

The study was grounded in an Indigenous methodology (Kaupapa Māori) and a participatory research approach. Data were collected from research documentation, community meeting and briefing notes, and interviews with community researchers.

**Results:**

The data analysis resulted in several key promising practices: *Kaumātua mana motuhake* (*kaumātua* independence and autonomy) where community researchers centred the needs of *kaumātua* in co-designing the programme with researchers; *Whanaungatanga* (relationships and connectedness) which illustrated how community researchers’ existing and emerging relationships with *kaumātua*, research partners, and each other facilitated the implementation process; and *Whakaoti Rapanga* (problem-solving) which centred on the joint problem-solving undertaken by the community and university researchers, particularly around safety issues. These results illustrate content, process, and relationship issues associated with implementation effectiveness.

**Conclusions:**

This study showed that relational factors are central to the co-design process and also offers an example of a braided river, or *He Awa Whiria*, approach to implementation. The study offers a valuable case study in how to translate, adapt, and implement a research-based health programme to Indigenous community settings through co-design processes.

**Trial registration:**

The project was registered on 6 March 2020 with the Australia New Zealand Clinical Trial Registry: ACTRN12620000316909. Prospectively registered.

**Supplementary Information:**

The online version contains supplementary material available at 10.1186/s43058-022-00374-3.

Contributions to the literature

- Research has shown that the culture-centred co-design of implementation of health interventions is important for Indigenous communities. Yet, there are few examples for communities and researchers of how such implementation works in practice.
- Frameworks for implementation and adaptation comprise many useful features that successfully guide co-designed implementation programmes. We found, however, that cultural and relational dynamics in concert with Māori community drive and agency were critical to managing the day-to-day real-world challenges of implementation.
- The findings add to the current literature by showing how using both Indigenous and Western knowledge can benefit communities, researchers, and the process of implementing health interventions.

## Background

Health inequities experienced by *kaumātua* (older Māori; Indigenous peoples) in Aotearoa, New Zealand, within the context of colonisation are well documented [1, 2]. *Kaumātua* (see the Glossary for English approximations of Māori terms) carry a significant burden in health, economic, and social inequities, despite cultural strength and resilience [3, 4], with calls for innovative and culturally based approaches to improving their well-being [5, 6]. Recent research identifies novel health interventions aimed at addressing such inequities [7, 8]. However, efforts focusing on the process for implementing evidence-based interventions within Indigenous communities are limited, particularly in Aotearoa [9]. Also, the benefits of Māori health provider initiatives and other Indigenous evidence are rarely reported in the literature [10]. Thus, groups who may benefit from implementing outcomes of research may miss out on opportunities to address health inequities.

Implementation science focuses on how best to implement an intervention, practice, or innovation that has benefitted one group of people, and adapt or modify the intervention with a different group or community setting [11–15]. The fundamental purpose of implementation science is to examine how to best support communities in accessing and adapting evidence-based interventions, programmes, or innovations that will benefit them [11]. Frameworks for implementation and adaptation aim to help researchers and communities to implement a given intervention successfully [12]. Two frameworks of interest to our study are the Consolidated Framework for Implementation Research (CFIR) [13, 16] and the He Pikinga Waiora (Enhancing Wellbeing) Implementation Framework HPW [17]. CFIR is a comprehensive framework that is frequently used in the implementation science literature, while HPW was developed from a Māori and Indigenous perspective and emphasises a participatory or co-design approach to engage communities and guide implementations processes which are advocated when working with Indigenous communities [18–21].

CFIR integrates 19 different models of implementation science and thus provides a comprehensive and inclusive framework that has been frequently used in implementation contexts [13, 16]. CFIR includes five elements: intervention, individuals involved, inner setting, outer setting, and process [13]. Intervention incorporates aspects of the intervention itself such as novelty, compatibility, relative advantage, supporting evidence, and whether the intervention has been adapted to local contexts [16]. Individuals are the implementers and their characteristics including cultural values, skills/experience, and affiliations. The inner setting refers to the organisation implementing the intervention and the level of managerial support. The outer setting encompasses larger economic, health, social, and political contexts in which the organisation operates. Process comprises the implementation methods and means [13].

HPW [17] is grounded in Indigenous knowledge, participatory approaches, and systems thinking and includes five elements: kaupapa Māori, community engagement, culture-centeredness, systems thinking, and integrated knowledge translation. Kaupapa Māori is a philosophy and methodology that centre Te Ao Māori (Māori worldview) to emphasise Indigenous epistemologies and knowledge [20, 22]. Although there are various community-engagement approaches [23], HPW emphasises participatory approaches with shared leadership and decision-making. Culture-centeredness ensures that communities adopting an intervention have agency in defining the problem and solution to their health issues. It also recognises that social structures framing these issues can only be transformed with community resources and through Indigenous self-determination [24]. Systems thinking recognises the multiple factors and levels that shape health issues and takes a holistic perspective to address the complexity of local contexts [18]. Integrated knowledge translation focuses on co-production with end users in the implementation process to enhance sustainability, community benefit, and effectiveness [25]. End users are the organisations and people who use research findings and interventions [13, 16, 17]. Terms such as “participatory”, “co-design”, and “co-production” within implementation science encompass a range of “partnership” processes. Such processes include community-identified issues, clearly articulated partnership structures, trusted community–researcher relationships, funding for the community group involved, and the guidance of the community’s cultural values and practices [21].

Research regarding implementation science with Māori communities is limited. This study describes the co-design and implementation processes used in translating and adapting an evidence-based peer-education programme for older Māori to new communities. The original study was premised on evidence that peer education offers older people facing social and well-being issues and transitions in later life [26, 27], emotional, informational, affirmational [28], and cultural support [29]. In Aotearoa, New Zealand, peer education is characterised by meaningful relationships between the “tuakana” as the senior experienced peer educator and the “Teina” as the junior inexperienced peer [30]. Here, the peer relationship is culturally based on Māori “tuakana–teina” (senior–junior) relationships [31] and differs from third-party and para-professional support, family, and community relationships [29]. There is great diversity in cultural communities in Aotearoa, New Zealand, including between Māori communities. In this respect, developing and implementing peer education within a given community needs to take an authentic co-design approach [13, 17].

The original research involved an evidence-based peer-education programme for *kaumātua* working through later-stage life transitions (e.g. loss of spouse and changing health condition) that was co-developed by a Rauawaawa Kaumātua Charitable Trust (Rauawaawa; Māori community organisation) and a group of University of Waikato researchers [32, 33]. The study found that the tuakana–teina (literally older sibling–younger sibling)/peer-education programme (TT programme) benefitted teina or “in-the-experience peers” [30] and enhanced tuakana (experienced peers) communication skills and this impacted positively on their social and cultural connectedness with their peers [34, 35]. The tuakana reported that their role strengthened their sense of cultural identity and well-being in the learning and positivity associated with gaining and sharing knowledge and enhancing a sense of self [34]. Teina identified enhanced social connections, self-efficacy, and informational support about health and social services. Finally, the TT programme was found to be cost-effective in addressing key health and social outcomes [34]. The value of the programme for kaumātua therefore warranted introduction to and adaptation by other community providers with their kaumātua.

The current research involved a team of four University of Waikato researchers (one Māori and one Pākehā co-PI, with two Māori and one Pākehā researcher) and two Māori Rauawaawa researchers (one co-PI and the lead community research [LCR]). The research used a co-design process with five Māori community providers so that they could adapt and implement the TT programme to meet the needs and preferences of their kaumātua and fit the local cultural practices or tikanga [36]. The purpose of this paper is to describe the factors and processes associated with community implementation and co-design of the TT programme, particularly considering Indigenous knowledge and perspectives about adapting and implementing an evidence-based programme. The following research question guided this study: What are the key implementation and co-design factors and processes that support Indigenous community providers to adapt the TT programme to meet *kaumātua*, cultural, and provider needs?

## Methods

The study used *Kaupapa Māori* (Māori methodologies) and community-based participatory research (CBPR) principles. *Kaupapa Māori* methodology prioritises Māori cultural worldviews and normalises Māori perspectives, principles, and practices [20]. CBPR prioritises community self-determination, community-identified issues, respect for different ways of knowing, community–researcher collaboration, and co-designed research [21]. Together, these approaches informed the “for-kaumātua-by-kaumātua” principle and strength-based approach (*mana motuhake*) used in the study. The HPW and CFIR implementation frameworks guided the process evaluation of what was important in the co-design (e.g. practices). HPW focused the evaluation on Indigenous knowledge, integrating cultural knowledge, and empowering processes within community partnerships [17]. CFIR focused the evaluation on the characteristics of the evidence-based programme, individuals involved, inner and out settings, and implementation methods [13].

The implementation of the evidence-based programme is guided by an advisory board comprising the Rauawaawa Kaumātua Charitable Trust Board of Trustees, the *kaumātua* they serve, and connectedness with the five Māori providers, their community researchers (CRs), and the *kaumātua* they serve. The research relationships are based on trust, care, and knowledge-sharing which ensured the integration of Kaupapa Māori and CBPR within the study.

### Research design

The end users were five Māori providers located in different rural and urban regions, their CRs, and the *kaumātua* they serve. The university researchers (URs) and Rauawaawa Kaumātua Charitable Trust (Rauawaawa) formed the lead research team and presented the outcomes of the original tuakana–teina/peer-education programme at the National Kaumātua Service Providers Conference in 2018. The potential for other community groups to be involved was offered at the conference to any who were interested should funding for a further study be awarded. Further conversations were held with the five provider groups who expressed interest. Once funding was secured (from Ageing Well National Science Challenge), the five agreed to take part beginning with preliminary face-to-face and Zoom conversations, document sharing, and orientation sessions at the *kaumātua* Service Providers’ National Conference held at Rotorua, Aotearoa, in November 2019. Further visits and Zoom meetings were held from December 2019 to March 2022.

The evidence-based programme was reviewed and adapted over a 9-month planning and co-design process with the service providers. Each service provider received resources to appoint a 0.5 FTE employee (i.e. CR ) to support the programme administration and research. The CRs started 3–6 months prior to starting the programme to allow for a robust co-design process. The research team developed initial documents and facilitated the co-design process. All processes and materials were then adapted, modified, and re-developed in consultation with the advisory groups and service providers. We then used the co-design process with each service provider to identify the key health and social issues particular to their community. Once these were defined, each provider created a resource kit (*kete*) to support the peer-education process. Although providers read the resource kit developed in the original research, each provider developed their own individual kit as a key adaption of the programme. Additional adaptions included the following: (a) changing images on orientation documents, (b) changing whakatauki (proverbs) on orientation documents, and (c) one provider changing the name of the programme. These adaptations did not change the functional elements of the programme.

**Fig. 1:**
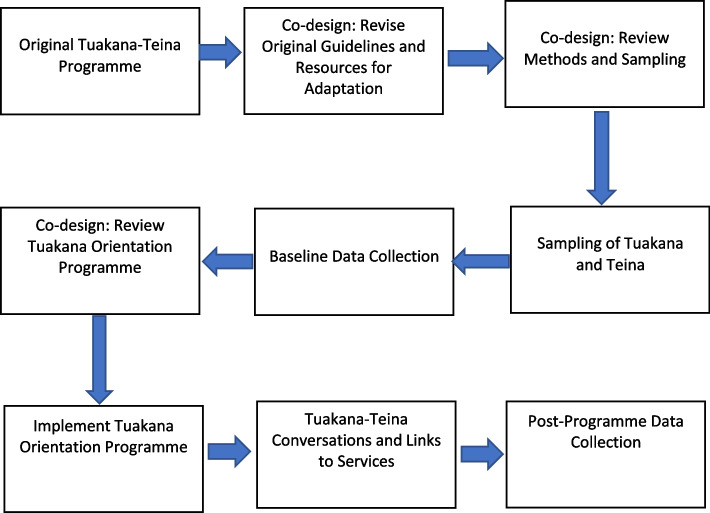
Tuakana–teina programme and co-design process

The CRs either ran or supported central processes within the programme: *kaumātua* recruitment, orientation sessions for participants, programme administration, and data collection. *Kaumātua* from each service provider participated in a Tuakana Orientation Programme (TOP) facilitated by the CRs with one university researcher and the LCR (both Māori) in support. In each community, four *kaumātua* served for 6 months as tuakana for six teina each, in six conversations focused on understanding teina needs and supporting them to gain access to needed health and social services. The outcomes of the programme for the kaumātua will be described elsewhere as the current study focuses on the implementation process and key factors. We believe the focus on process is critically important for working with Indigenous communities. See Fig. 1 for the overall co-design process.

### Data collection

Ethical approval for data collection was provided by the University of Waikato (HRECHealth2019#81). First, data were collected from September 2019 to December 2020 from several sources (no research activities for four months due to COVID-19). These included notes (minutes) from meetings with each Māori provider group. Central tasks included sharing information, resources, documents, and templates and talking about data collection, measures, ethics, and contracts. These meetings usually included the lead researchers asking three questions of the providers: “Where things are at for you with the project? What are your current challenges and successes? What support do you need from us?” Where possible, data were collected from email and in-person conversations between the researchers and individual CRs. These data comprising 45 meetings were combined into one document (28 pages, single-spaced) for thematic analysis.

Second, semi-structured joint interviews (30–60 min; see Supplemental file 1) were conducted by the Māori CR from the lead research team and the Māori project manager (both experienced in qualitative methods), with seven Māori CRs from four community providers (one group could not take part due to workload). The conversation focused on the CRs’ experience in the co-design process, with the interview guide comprising six questions related to their co-design experience (see Supplemental file 1). Ethics documentation was outlined before each interview, with consent to participate and record being audio-recorded. The interviews were approximately 1 h and completed over 4 months in 2020. Due to COVID-19 restrictions, these were held via Zoom©. The audio recordings were transcribed and resulted in 30 pages of single-spaced text. The conversations followed kaupapa Māori practices, starting with a karakia (prayer) and pepeha (formal introduction/greeting) and ending with a karakia [31]. This procedure helped to build whanaungatanga (relationships) and make Iwi (tribal) and other connections between participants.

### Data analysis

The interview transcripts and document texts were coded using thematic analysis [37, 38]. Four researchers (3 Māori, 1 Pākehā) coded the raw data for emergent themes across the transcripts. Therefore, we first individually and collectively identified and (re)interpreted the patterns of meaning within spoken text in a fluid process of conversations among the coders and then compared with the documents. Second, we used the HPW [17] and CFIR [13] in framework analysis [39] to guide the interpretation of the initial themes. Together, these processes led to decisions about the final themes. Finally, to enhance trustworthiness, the results and draft paper were shared with the CRs and Advisory Board for guidance and reshaping. A completed COREQ checklist is provided (see Supplemental file 2).

## Results

The three themes detailed in this section (see Table 1) show how the implementation and co-design processes supported the community providers in adapting the TT programme to meet *kaumātua*, cultural, and provider needs. The three main themes were *Kaumātua mana motuhake*: *Kaumātua* autonomy, *Whakawhanaungatanga*: relationships, and *Whakaoti Rapanga*: problem-solving. Each theme comprises two or three subthemes. In keeping with Māori culture-centred approaches, we identified *whakatauki* (proverbs) as complementary value statements for each theme. *Whakataukī* offer knowledge or wisdom that guides choices and actions [40, 41]. The information in parentheses at the end of quotes refers to the data sources. Within the quotes, the parentheses add context/interpretation when a quote has a missing word or Māori word.

**Table 1:** Summary of themes with illustrative participant quotations

Theme	Subtheme	Description	Illustrative participant quotation
**1.*Kaumātua mana motuhake:*Kaumātua autonomy** **Proverb**: *Nō te mea rā ia he rākau tawhito, e mau ana te taitea i waho rā, e tū te kōhiwi* For it is certain that in a very old tree the sapwood is on the outside and heartwood stands firm	1.1 *kaumātua* centredness of TT programme.	The kaupapa of the programme was centred on *kaumātua* wellbeing and perspectives.	• “You had to get the *kaumātua’s* permission, to see if they were okay about it and ask them “what is important to you” (2b-29). • “We need to remember who we’re focusing on and it’s *kaumātua*” (4b-4a)
	1.2 *kaumātua* centredness of implementation	Implementation was based on *kaumātua* needs not research needs.	• “This *rōpū* (group) said ‘this is the way’ that they wanted to go. That’s what I liked about it (the programme)” (4b-45c). • “We need to make sure that they have a pathway, a safety net to support that *kaupapa* (purpose) for the *kaumātua*” (4d-135b).
**2.*Whakawhanau-ngatanga:*relationships** **Proverb**: *He kura te tāngata* The human being is precious	2.1 CR–kaumātua relationships	The importance of building and nurturing relationships with kaumātua	• “It takes a little time to build rapport with our *whānau*. It’s not a matter of just going, ‘And this is what you know’ –that takes time, and once you build the trust then you can start introducing ideas” (1b-83). • “Once you got into it [talking] it was really good; [*kaumātua*] were quite receptive [to] a project about them, [where] they were the main focus of this whole research. Some of them felt it was quite important” (2a-17a).
	2.2 CR–UR relationships	The importance of building relationships with the lead researchers.	• “When things haven’t gone so good I’ve been able to talk to someone like [named URs] and let them know just about where we’re at—then we’ve been able to carry on” (4b-45). • “We asked [named UR] to come and talk to the kaumātua—this wasn’t part of the plan. [UR] came and talked to the *taumata* (decision group of *kaumātua*) about the project and that wasn’t on the schedule, that was out of the box” (2a-153).
**3.*Whakaoti Rapanga:*problem-solving** **Proverb**: *He moana pukepuke e ekengia te waka* A choppy sea can be navigated.	3.1 Flexibility of implementation strategies	Providing structure for programme implementation, and allowing for flexibility.	• “[the resource *kete*] template was perfect because it gave us the structure of what they were looking for. And we opted to basically stick with their template and add all the services and health providers that were in our area; it was great” (3b-94). • We’ve loved that we could change some of the stuff and make it—[locally] centred” (3a-65)
	3.2 Adaptability to *kaumātua* life factors	Compassionate management of *kaumātua* challenges	• “We’re not going to be the only ones that have people with literacy issues, and is there tricks and tips that the researchers have that we [could] add into the *kete* (resource kit) for the tuakana, about supporting people with literacy” (2b-111). • “One of our [*tenia*] had one of her *mokopuna* (grandchildren) staying with her, who was on synthetics and they don’t want to say because they’re embarrassed, and they don’t want to share that stuff about their families. So, there’s going to be some real sensitive kōrero (talking). How do you balance that?” (1a-129c).
	3.3 Response-ability to external factors	Adjusting to the challenges of the COVID pandemic	• We just came out of COVID, when we started our big first intake. So, of course, they were all happy chappies because they’ve been stuck at home, but some of them weren’t quite so happy [due to fear of illness]” (2a-35). • “It was a bummer about COVID, but we’ve been able to be flexible. We made the agreement that even though we’ve [used] the funding and we’ve employed somebody else to look after [the CR] we’re going to manage that going forward even though we will go past the contract time. That was really good to be able to do that” (2b-25a).

### Theme 1: *Kaumātua mana motuhake*: Kaumātua autonomy

The first theme describes how the provider-based CRs centred *kaumātua* needs in co-designing the programme implementation with researchers. This theme’s *whakataukī* was: *Nō te mea rā ia he rākau tawhito, e mau ana te taitea i waho rā, e tū te kōhiwi*; For it is certain that in a very old tree the sapwood is on the outside and heartwood stands firm [41]. This proverb refers to the advice and support of *kaumātua* in leading young warriors in defending the tribe. In regard to this study, it speaks to the centrality of *kaumātua* in the successful co-design of a programme focused on *kaumātua* well-being. The two subthemes highlight the value of *kaumātua mana motuhake* (*kaumātua* independence and autonomy) in the *kaumātua* centredness of the TT programme and CRs’ implementation activities.

#### Subtheme 1.1: *Kaumātua* centredness of the TT programme

This subtheme captures CRs’ view of the TT programme’s *kaupapa* (focus/purpose) as *kaumātua*-centred. The focus on *kaumātua* well-being resonated with the CRs with one extolling, “the concept of this research is awesome” (1a-111a). Another CR suggested programme benefits for *kaumātua* beyond those taking part because, “The issues that face *kaumātua* are universal … so, it’s going to be very helpful” (3a-98a). The CRs recognised issue commonality for *kaumātua* across the different providers and the potential of the programme to help meet those needs.

One group of CRs anticipated the TT programme would benefit future generations of Māori because it was “sowing the seeds, *rangiatea* for the next *mokopuna* (grandchildren), the next generation” (4d-145) “when they come through to become *kaumātua*” (4a-147). Here, the CR referenced “*Rangiatea*” (4b-145), the departure location for Māori migration from Hawaiiki to Aotearoa [41]. The CR invokes the cultural significance of that event to suggest the potential of the TT programme to be a dispersal point for something of lasting influence.

#### Subtheme 1.2: *Kaumātua* centredness of CR implementation

The second subtheme captures how *kaumātua mana motuhake* was evident in CRs’ activities implementing the TT programme to support *kaumātua mana motuhake*. They described *kaumātua*-centred implementation activities aimed at engaging and supporting *kaumātua*. Such activities included “live workshops [that] brought them together, and we went through [the TT programme]” (2a-17a) and talking one-on-one: “I went to see two *kuia* (older women) the other night. [The kuia asked] ‘Ah, what’s that for?’ ‘What does that mean?’ You sort of let them have their kōrero (talk)” (1a-111c).

The CRs also fed back on data collection instrument questions (minutes 04-0820). As one commented, “it’s having a good think about the questions, as to how relevant it is for [our *kaumātua*]” (2a-31). In these instances, the CRs used different methods to “enhance the *mana* (standing) of *kaumātua*” (4d-135a) and provide pathways to *kaumātua* understanding the TT programme in their own way.

The CRs allowed time and opportunities for *kaumātua* to ask questions and to get to know the CRs. As one CR noted, “it was the unknown; [*kaumātua*] were very unsure” (2a-17a) about the research, programme, and their involvement. Another suggested asking *kaumātua* “What is important to you?” (minutes 02-141019). In focusing on the needs of *kaumātua*, CRs engaged in Māori culture- and *kaumātua*-centred processes that enhanced *kaumātua mana motuhake* and the programme implementation*.*

CRs appreciated being able to adapt the TT programme to meet *kaumātua* needs. For one CR, it was important that the *kaumātua* group she worked with was able to choose how the programme would work for them (4b-45c). Similarly, another said “What has been really good is that flexibility …around the whole programme; how it’s going, how it’s being run” (1b-73). Such flexibility suggests that the TT design was responsive to the needs of local *kaumātua* and the CRs, while also maintaining its *kaupapa*: to enhance *kaumātua* capacity to meet *kaumātua* needs. The flexibility empowered CRs during implementation and supported *kaumātua* and their *mana motuhake* in the programme.

### Theme 2: *Whakawhanaungatanga*: relationships

The second theme centres on relationships within the co-design process. These relationships included CRs’ existing and emerging relationships with *kaumātua,* research partners, and each other. The theme’s *whakataukī* was *He kura te tāngata*; The human being is precious (34). This proverb highlights the intrinsic value of people and also “the contribution of each person to the well-being of the group” (p91) and connections between people within the group. In the case of the TT programme, the people included *kaumātua,* CRs, service providers, and researchers and the relationships and connectedness between them. The subthemes illustrate the centrality of CRs’ relationships with *kaumātua* and research partners.

#### Subtheme 2.1: CR–*Kaumātua* relationships

The first subtheme explains the developing relationship between CRs and *kaumātua* in the co-design process. CRs emphasised “building” (1a) and “nurturing” (4d) relationships with *kaumātua* as the first step of engagement and implementation and ensuring adequate time for this. This process involved CRs prioritising the relational over the programme’s instrumental dimensions. For instance, one CR emphasised “rapport” which suggests relational characteristics of closeness and understanding between the CR and *kaumātua*. Another CR distinguished between her knowing *kaumātua* in Māori cultural settings, such as *marae* (community meeting place) and *tangihanga* (funeral), and how “you get to know [*kaumātua*] on a different level when you’re working directly with them” (1a-111b) in the TT project. The phrase “get to know” process suggests a temporal component to new developing relationships between the CR and *kaumātua*.

CRs getting to know *kaumātua* helped build confidence in the TT programme and its focus. For instance, one participant stressed that talking through issues with *kaumātua* helped *kaumātua* to realise that they were the main focus. Talking through the TT programme with *kaumātua* helped them to understand the relevance of the programme for them.

#### Subtheme 2.2: CR–UR relationships

The second subtheme features the CR–university researcher (UR, *kairangahau*) relationship in the co-design process. CRs’ comments emphasised connectedness, responsiveness, and flexibility in their relationships with the lead community agency (Rauawaawa) and URs. Connectedness of CRs with URs appeared in discussions about the programme and addressing challenges in the early phases. CRs in one group said they were “really lucky because we already had a relationship” (2b-131) with the lead community group and URs and a sense of what the TT programme was about. Other CRs who were new were initially overwhelmed with the idea of research and the programme as well as the introductory information: “The Rauawaawa and the University knew exactly what they were doing, but all of us providers were like, ‘ahh!’” (2b-25). This comment captures the stress experienced by CRs in early-stage implementation. Although they understood and valued the TT programme, operationalising implementation at the local level was initially challenging for CRs. However, CRs commented on the ease of access to and responsiveness of the URs to their needs throughout the implementation. One illustrative comment was: “They’ve always been available. They’ve always made everything clear” (3b-35a).

Such statements highlight how CRs positively experienced their interactions with URs particularly during the early stages of implementation. The CRs were able to communicate their concerns to URs and the URs responded positively to requests for clarification and support. In sum, the qualities of the community implementers and Rauawaawa, and university researcher relationships, facilitated a collaborative approach to implementing the TT programme.

### Theme 3: *Whakaoti Rapanga*: problem-solving

Theme 3 centres on the joint problem-solving undertaken by CRs and URs. This theme’s *whakatauki* was: *He moana pukepuke e ekengia te waka—A choppy sea can be navigated.* This *whakataukī* shows the *waka* (double-hulled boat) as a vessel designed for long-distance sea journeys and to be navigated by people and is a metaphor of collective decision-making and acting together in response to changing conditions. The subthemes concern flexibility of implementation strategies, managing the *kaumātua* life factors, and dealing with external events.

#### Subtheme 3.1: flexibility of implementation strategies

This subtheme concerns factors associated with the flexibility of CRs in implementing the programme and adapting processes to meet *kaumātua* needs. Processes discussed centred on recruiting and matching *tuakana* and *teina* and responding to emerging *kaumātua* needs.

Recruiting and matching were closely linked processes. CRs began by identifying the greatest needs of *kaumātua* in their area. These included but were not limited to, loneliness, isolation, loss of driver’s licence, access to transport (to doctors), strength and balance, elder abuse (financial, emotional, and mental), diabetes, loss of independence, dementia, emergency housing, chronic health conditions, rural *kaumātua* and limited access to services, need for own cultural connectivity (minutes 11-191219). Knowing which agencies offered appropriate support was the foundation for CRs creating a resource *kete* of local services. However, as one CR noted, “The worst thing we want to do is refer our awesome *kaumātua* to *hako* [disrespectful] people [in other providers]” (minutes 02-141019). Thus, knowledge of local support services was critical for CRs and their resource *kete* which the researchers encouraged them to “adjust to suit [their] *rohe* [region], *kaumātua* and their needs” (minutes 02-141019).

At CR–UR meetings, CRs sought help from the research team and the other CRs about strategies for recruiting *teina* and matching with them with the appropriate *tuakana*. One recruiting strategy offered was being ready to interact with *kaumātua* and having “in hand ready for the potential *tuakana* or *teina*” the information and consent documents (minutes 02-141019). When asked about matching *tuakana* with *teina*, the lead CR suggested that CRs, “Ask *kaumātua* about their interests [which] could be used to identify *kaumātua* needs and to match *tuakana* with *teina*” (minutes 02-141019). Another suggested using a sheet with questions “What is important to you? [need] What is your area of interest? [matching]” (minutes 02-141019). Responses were then collated as a spreadsheet to help the CR match the *tuakana* and *teina*. One broader strategy sought support from *kaumātua* service providers to promote the TT programme. For instance, this CR started by “organising a *hui* [meeting] with the managers, [of] two *hauora* [health service agencies] … to give a group [*kaumātua*] *kōrero* [presentation; conversation]” (1a-65). Sharing their strategies helped facilitate CRs’ learning from the lead CR’s previous experience as well as each other. CR comments offered examples of responses to implementation flexibility: “I think the co-design with the team, and the research[ers] … the assistance and support provided from the researchers … has been great.” (1a-31). Such comments highlight the dynamics of implementation in the relationship between structure and support provided by the research team and the adaptability of programme implementation within local contexts.

In summary, flexibility was central to CRs’ successful implementation, as they navigated the social, cultural, and geographical dynamics of recruiting and matching *kaumātua* in their own areas. Flexibility was also central to CRs negotiating *kaumātua*-related factors that impacted programme implementation.

#### Subtheme 3.2: fitting to *kaumātua* life factors

This subtheme centres on emerging individual, social, *whānau*, and health factors identified by CRs as impacting *kaumātua*. These factors were sometimes already known to the CRs in their role within the agency, and at other times emerged from data collection procedures. One such issue was literacy. The first stage of data collection asked *kaumātua* to complete a questionnaire; it was here that CRs identified some illiteracy among the *kaumātua*. This prompted changes in administering the questionnaire. For example, one CR collected data one-on-one after discovering in the process that she had “five that cannot read and that impacts on them in general, because they don’t understand when they go to the doctor - they can’t read their prescriptions” (2a-35b). Thus, through the research activities, the CRs identified literacy issues and changed the research process to match and also recognised the wider implications for *kaumātua* in accessing services; they then sought help from the research team for resources to include in the resource *kete*.

Other factors emerged early in the process. CRs asked questions about how to best support *teina* and *tuakana* when sensitive issues such as “elder abuse incidents” (email-2/20; minutes 120220) arose. The providers did not have staff working in these areas, and they were reluctant to refer to “strangers” at another provider (email-2/20). In conversation with the URs, it was decided that Rauawaawa staff would provide support.

One CR talked about wanting to reassure *kaumātua* about the confidentiality of people’s lives where “some stuff should stay with [*tuakana* -*teina*] … some stuff should stay with our *whānau*” (1a-129a). She was concerned about *tuakana* listening to *teina* talk about sensitive issues (e.g. sexual abuse, drug use) without becoming involved themselves and about not exposing the *teina*’s lives. This CR was concerned about finding ways to balance supporting the *teina* with life issues already known to her and trusting the *tuakana* to deal with it should it arise in the conversation. She later acknowledged “If they’re [*teina*] going to be honest and they feel comfortable with their *tuakana*, it will come out” (1a-177c) and thereby addressed her original concern.

Although the *tuakana* orientation programme included a “what to do” when *teina* raised sensitive or urgent issues, follow-up “booster sessions” were held with *tuakana.* Here, the URs focused on trust and how *tuakana* could share their own stories with *teina* as a way to build connections without taking on the *teina*’s issues (meeting-09/2020). These processes endorsed *tuakana* knowledge and self-efficacy in their implementation of the programme. This subtheme also shows how the climate of trust combined with programme flexibility and skill building supported problem-solving and the implementation itself.

#### Subtheme 3.3: response-ability to external factors

This subtheme focuses on external factors that impacted the programme implementation. The most critical of these was COVID-19 which resulted in a continuous lockdown from 23 March to 13 May 2020 and suspension of the research for 4 to 6 months. The resulting challenges with co-design and implementation centred on restarting the programme in the context of uncertainty among *kaumātua* and their *whānau*. Some CR comments reflected challenges in restarting after the COVID lockdown: with the *tuakana* being “a bit reluctant to engage, and for a couple that are favourable, it’s their *whānau* [family] that are reluctant for them to engage because they’re in that vulnerable group” (1a-45). The concern about COVID for older Māori was pervasive among the providers as well as *kaumātua* and their *whānau*. This resulted in CRs having multiple conversations with individual *kaumātua* and their *whānau*. Although this increased the workload, it also demonstrates how CRs adapted to the changed environment and emerging uncertainty.

The suspension also impacted the programme funding, and for at least one provider, this meant losing their original CR. Providers in this situation did not have the internal resources to retain the CR whereas other providers were more fortunate and were able to absorb the financial impact of the lockdown. This suggests that some providers were more financially resourced and, as a result, more able to exercise choice. It also demonstrated commitment to the programme. As one CR summarised the situation. “excellent communication, crappy time of the year with COVID. Yeah, we’re super excited to get it started from our end anyway” (3b-35b).

In summary, this theme highlights the flexibility within the programme itself and the support of the research team for CRs. It also highlights the response-ability of CR to emerging *kaumātua* needs and the impact of COVID on the programme.

## Discussion

This study explored the co-design and implementation processes used in translating and adapting an evidence-based peer-education programme for older Māori to new communities. The guiding research question was as follows: What are the key implementation and co-design factors and processes that support Indigenous community providers to adapt the TT programme to meet *kaumātua*, cultural, and provider needs? The key themes are discussed in relation to features of the HPW and CFIR implementation frameworks.

### Relational dynamics

The results highlighted the importance of relational dynamics in the co-design processes [42]. The relationships between CRs and kaumātua and CRs and the research team started with existing trusted relationships (e.g. previous work together) or provider relationships with the lead community partner. These foundational relationships supported the CRs in leading and adapting the programme to align with cultural and organisational values, meet *kaumātua* needs, and respond to situational (e.g. *kaumātua* literacy) and external factors (e.g. COVID-19). Furthermore, the CR–kaumātua and CR–UR relationships were based in shared values (e.g. Kaupapa Māori, care for kaumātua), respect for each other’s perspectives and contributions, and mutual engagement facilitated by a participatory approach. The participatory and relational approach resulted in CRs’ partnering in the co-design of research materials (e.g. resource kete, questionnaires), and leading in successfully recruiting kaumātua, running the Tuakana/Awhina and Teina/Kaumātua Peer Educator Orientation Programmes, and managing the peer-conversations.

The relationships were developed and maintained within a climate of mutual learning and problem-solving [13, 16]. The regular open communication, individual and joint CR and UR sessions facilitated collaborative responses to emerging internal (e.g. *kaumātua* literacy) and external factors (e.g. effects of COVID-19 lockdowns). These dynamics provide the foundation for an overall successful implementation in trying conditions. Together, these are examples of the HPW processes of culture-centredness, community engagement, and integrated knowledge translation practices that are central to programme adaptation and implementation [9, 17]. They illustrate the importance of building trusting relationships in order to engage with partners effectively and ensure a programme and process that follows cultural protocols. The original CFIR framework does not directly consider relational components; however, a new CFIR 2.0 identifies the importance of members of the inner setting having strong partnerships/relationships with those in the outer setting to support implementation [43]. Additionally, it identifies teaming as a key part of the implementation process. Both of these elements are consistent with the current findings.

These relational processes supporting implementation are consistent with growing research about implementation effectiveness in Indigenous communities [44, 45]. Successful implementation projects of evidence-based interventions in Indigenous communities are grounded in participatory community engagement principles [44, 46]. Blue Bird Jernigan and colleagues [44] reviewed five successful implementation cases with Indigenous communities in the USA and all were grounded in community-based participatory research. This type of community engagement allows for building strong and trusting relationships among researchers and community members and organisations to facilitate the implementation process.

### Programme fit: identification and self-determination

From the CFIR perspective, identification with and self-determination of an intervention by the implementing individuals and organisations were important factors for success [13, 16]. The CRs identified with the TT programme because it aimed to benefit *kaumātua* they knew and worked with. One additional programme strength for CRs was that the lead community agency (Rauawaawa) who had previously helped developed it was now supporting them with implementation. This recognition implied that the programme itself was culturally strong. Another strength of the programme was its openness to provider agencies in adapting the programme to fit the local context. Finally, a related strength was with the CRs themselves; they valued and readily led and adapted the programme, resources, and strategies to meet emerging challenges and needs of kaumātua. This CR agency and adaptation revealed self-determination as being foundational to successful implementation [17]. Together, these examples demonstrate success factors within CFIR including respect for source, quality, strength, and adaptability of the programme and important individual characteristics including implementer knowledge, agency, and self-efficacy [13].

Programme fit, and particularly the ability to adapt the programme to enhance fit, is consistent with implementation research in Indigenous communities [44, 47]. Blue Bird Jernigan et al.’s review of the five implementation cases all provide adaptations of the original intervention to fit the local cultural context [44]. Coupled with the relational processes through strong community engagement, adaptation enables local community members to determine what aspects of the intervention work and thus increase the likelihood of a successful implementation process and outcomes [44]. These results and findings from other studies are also consistent with the HPW framework’s emphasis on community engagement and culture centeredness to enhance cultural fit through programme adaptability [17]. Given this research base and the current findings, we strongly suggest that when implementing with Indigenous communities, researchers and practitioners engage with community providers and community members early in the selection of an evidence-based programme. The community should have self-determination in that selection. Furthermore, the promising practices may offer direction for the implementation processes; at the very least they can be used to have a conversation with community partners to best determine adaptations, programme fit, and implementation processes.

## Limitations

This evaluation was undertaken mid-way through the implementation with a view to the final evaluation to be undertaken at the end. Further evaluation will be undertaken at the end of programme implementation to compare different community settings. Thus, a key limitation is that we do not have direct evidence as to the impact of the adaptations and the implementation process undertaken in this study. However, we do believe that the co-design process identified in this process that led to strong relationships and a supportive adaptation process are promising practices for implementing evidence-based programmes with Indigenous communities. This argument is supported by the extant literature showing the benefits of community engagement during implementation with Indigenous communities.

As is often the case with qualitative research, participants’ numbers are low and the data is rich. The interviews were conducted by two Māori members of the wider research team who, although known of by the CRs, had no previous contact with them prior to the evaluation. Finally, this implementation occurred during the COVID-19 pandemic which impacted how the providers and community researchers continued the study while also meeting the needs of the often vulnerable kaumātua taking part.

## Conclusions

This study showed that relational factors are central to the co-design process. Co-designed implementation centres on partnering with those most impacted by an issue, to address that issue [21]. Within these partnerships, relational factors included specific actions such as open communication, shared decision-making and problem-solving, CR-led implementation, enacted shared values and aspirations that supported kaumātua, and the flexibility of the programme itself (*c.f.* [17, 21, 44]). Programme flexibility is considered a “relational factor” because it is pivotal in meeting community needs in ways that work for those most affected [17].

The study may also offer an example of a braided river, or He Awa Whiria, approach to implementation. This approach recognises the value of distinct Māori and Western streams [48] in creating a “workable whole” ([49], p18). Braided Māori and Western streams may be evident in the joint implementation by the Māori community providers, Rauawaawa as the lead Māori community agency, and the URs (Māori and Pākēha). The study was founded on Māori values and practices and used traditional Western methods (e.g. focus groups and meeting documents). Similarly, the study drew on the principles of the HPW [17] and CFIR [13] implementation frameworks. Furthermore, the study was a mid-way evaluation and relied on a mix of data collection (e.g. documents) and in-the-moment reflection by CRs conducted within a forum guided by Māori communication protocols and practices [35]. In sum, the study shows the value of collaboration where community partners’ self-determination and autonomy and Māori values and practices are embedded in the co-design. This is particularly relevant within international contexts where Indigenous peoples and concepts are not always supported to lead and determine research [17, 44].

Future research would likely benefit from capturing partners’ voices along the way with culturally-grounded methods (e.g. hui, wānanga, photo diaries, podcasts) with decisions being jointly developed by the implantation partners. In conclusion, the study offers a valuable case study in how to translate, adapt, and implement a research-based health programme to community settings through co-design processes. In privileging co-design founded on *Māori* values and practices, within a relational climate of trust, the intervention was implemented and adapted for local Māori *kaumātua* by local *Māori* providers. This model of community–university research partnership offers other communities, and especially Indigenous communities, practical steps to working with researchers to co-design programmes to meet their own identified needs.

## Supplementary Information

**Additional file 1.** Interview Questions.

**Additional file 2.** COREQ (COnsolidated criteria for REporting Qualitative research) Checklist.

## Data Availability

The datasets used and/or analysed during the current study are available from the corresponding author on reasonable request.

## Abbreviations

CFIR: Consolidated Framework for Implementation Research
CR: Community researcher
HPW: He Pikinga Waiora
UR: University researcher

## Glossary

Aotearoa: Māori name of New Zealand
he awa whiria: Braided rivers
hui: Meeting or meetings
iwi: Tribal group
kairangahau: Researcher
karakia: Prayer
kaumātua: Older people
kaumātua mana motuhake: Kaumātua independence and autonomy
kaupapa: Task or focus
Kaupapa Māori: Māori methodology located within Te Ao Māori
kete: Kit, basket
kōrero: Talk
kuia: Older woman
mana: Standing, status
mana motuhake: Identity, autonomy
Māori: Indigenous peoples of Aotearoa New Zealand
marae: Community meeting place
Pākehā: New Zealander of settler heritage
rohe: Region
Te Ao Māori: Māori worldview
tangihanga: Funeral
teina: Junior to tuakana (the older or less experienced)
tikanga: Cultural practices and protocols
tuakana: Senior to teina (the younger or less experienced)
tuakana–teina: Older and younger, same-sex sibling or cousin relationships
whakatauki: Whakatauki (proverbs)
whakawhanaungatanga: Making connections and establishing relationships
whānau: Closely connected kin group
whanaungatanga: Relationships and connectedness
whakaoti rapanga: Problem-solving
