# Supplementary material for: Adaptation and implementation processes of a culture-centred community-based peer-education programme for older Māori
Source: Implement Sci Commun. 2022 Nov 24;3:123. doi: 10.1186/s43058-022-00374-3 (PMC9694883; doi:10.1186/s43058-022-00374-3)
Supplement: Supplementary file 1 — Additional file 1. Interview Questions. [file 43058_2022_374_MOESM1_ESM.docx]

**Supplemental File 1: Interview Questions**

1. What do you think is working well overall in the Tuakana-Teina project?

1a. prompt eg.: What would you like to see continue?

1. What do you think could be improved in the overall project?

2a. prompt eg.: What would you like to have/see more of / less of?

1. What does ‘co-design’ mean to you in practice? .... in this project?
2. How well are the university and community researchers co-designing this project?

4a. prompt: What’s an example of co-design that has worked particularly well for you during this project?

4b. prompt: What’s an example of co-design that could change/be improved for you during this project?

1. How has the working relationship between the university researchers and yourselves been, from your experience so far?

5a. prompt: What would you like to see more or less of?

1. How successful do you think this project will be?

6a. prompt : In what ways? What might success look like to you?

1. That’s the end of our questions, what questions would you like to ask us about the project?
